# Medico-Legal Notes

**Published:** 1891-09

**Authors:** Henry A. Riley

**Affiliations:** New York


### MEDICO-LEGAL NOTES.

By HENRY A. RILEY, Esq., New York.

### INCREASE OF CRIME AND THE DEATH PENALTY.

A recent writer in the *Judicial Review*, says that murder has increased to an alarming degree in those countries where the death penalty has been abolished. In France, from 1828 to 1884, murders increased from 197 to 234 a year; infanticide, from 102 to 194 ; blows and assaults, from 8,000 to 19,000 ; robberies, from 9,000 to 33,000 ; and that in 1885 crime was still increasing.

In Belgium, murders increased in a frightful manner whenever the knowledge of the abolition of the scaffold spread among the masses. In Prussia, where for many years there had been no executions, murders increased from 242 in 1854, to 518 in 1880. In Switzerland, where capital punishment was abolished in 1874, murders increased in five years in the proportion of seventy-five per cent.

This startling increase is in some slight degree explained by the fact of the considerable increase in the population of these countries ; still, it is difficult to account for the remarkable change, except on the theory of the deterring effect on criminals of the death penalty.

In this country most of the States inflict the death penalty, but in some of them there is a practical abolition of executions, even when the law authorizes them. In Kansas, for instance, the statute says that the Governor "may" fix a day for execution, and the result is that there has been no execution for years, while the prisons are full of convicted and sentenced murderers. The Governors say that if the Legislature wishes to have the death penalty inflicted, it can easily change the word "may" to "shall," and in such case they would promptly issue the warrants.

In New York, the attorneys for convicted murderers have discovered a way of indefinitely prolonging their lives by appealing from the State courts to the Federal tribunals. It seems possible to take such appeals on frivolous and purely technical grounds, and the crowded calendars of the Federal courts prevent their consideration and decision for long periods.

---

### MUST PHYSICIANS ANSWER CALLS ?

According to a newspaper report, a New Haven physician was fined not long since $10, because he did not answer a call in an urgent case. It is very doubtful if such a case actually occurred, as it seems quite clear that there is no legal liability on the part of physicians to give their services to every one who may call for them. If it were so, the lives of some popular and trustworthy physicians would be a burden too great to be borne.

The principle of the common carrier in law, which requires railroad companies to accept and transport all passengers and freight offered them at regular rates, does not have any application to physicians so as to force them to give their services to all comers.

It is just that railroads should be under such a duty, as they are public corporations and receive valuable franchises from the State for which every citizen has a right to demand an equivalent.

Perhaps the point has never been directly decided by a court of high jurisdiction, but in a recent New York case where a physician was charged with malpractice, one of the defenses was that he was treating the patient gratuitously. The judge, in charging the jury, used this language:

> He may decline to respond to the patient unable to compensate him, but if he undertakes the treatment of such a patient he cannot defeat a suit for malpractice, nor mitigate a recovery against him upon the principle that the skill and care required of a physician are proportioned to his expectation of pecuniary recompense.

This is practically a decision of the point.

---

### ARE PUGILISTS ARTISTS?

The recent pugilistic encounter in which an Australian, named Slavin, was victorious, has had the curious result of drawing attention to a violation of the Contract Labor law. It is now claimed that the bargain made by Slavin and Mitchell to exhibit themselves in pugilistic encounters through the country was a violation of the law, which only allows "artists" to be exempt from its provisions.

A letter from an individual who resented their coming to this country on such a brutal errand, when a person is prohibited from bringing a servant into this country, was the means of calling the attention of the Commissioners of Immigration to the matter. The men have now both returned to England, so that nothing can be done to them for breaking the law, but the speculator in pounding, who brought them here and then failed to carry out his agreement, is liable to a heavy fine. He can only escape if the matter is pressed by proving that pugilists are "artists," and an effort to do this will be watched with interest. It may occur to him that as barbers and other like individuals are occasionally styled "professors," it is no great stretch of the imagination to conceive of the representative of the "fistic art" as an artist. One result of the matter will probably be, that native pugilists will be to some measure protected against the competition of foreign sluggers, and that our own citizens will have most of the honor, glory and money of the prize ring.

### AN INFANT CRIMINAL.

THE Omaha *World-Herald*, of a recent date, has this interesting editorial item: "There is a certain way to make criminals, just as there is a certain way to make houses or clothes, or anything else you are minded to mention.

"One of the best and quickest ways to make a criminal, is to charge a baby of seven years with grand larceny, and lock her up in the police station. Tillie Schuster is said to have stolen $50. Perhaps she did; but if she did, she did not know the difference in value between that and fifty cents. Ask any child of seven whether he would rather have one dime or nine pennies, and if he sees them in your hand he will choose the nine pennies.

"If Mr. Wicksworth thinks Tillie Schuster stole his money, there are other ways of getting it back or finding out where she has put it or how she has destroyed it, than by arresting her and putting her in with criminals. That was a crime worse than larceny."

### A SUIT BY SIR MORELL MACKENZIE.

SIR MORELL MACKENZIE has just brought suit for $10,000 damages against the Sodene Mineral Spring Co., claiming that his name was used without authority in the advertisements printed by the company. A temporary injunction has been granted, and the trial will be held after a time. Sir Morell charges the defendants with treading upon his professional reputation.

